# Numerical Evaluation of Image Contrast for Thicker and Thinner Objects among Current Intraoral Digital Imaging Systems

**DOI:** 10.1155/2017/5215413

**Published:** 2017-04-09

**Authors:** Oyunbat Dashpuntsag, Midori Yoshida, Ryosuke Kasai, Naoki Maeda, Hidehiko Hosoki, Eiichi Honda

**Affiliations:** ^1^Department of Oral and Maxillofacial Radiology, Tokushima University, Tokushima, Japan; ^2^Radiological Center, Tokushima University Hospital, Tokushima, Japan

## Abstract

The purpose is to evaluate the performance of current intraoral digital detectors in detail using a precise phantom and new method. Two aluminum step wedges in 0.5 mm steps were exposed by two photostimulable phosphor plate (PSP) systems—one with automatic exposure compensation (AEC) and the other without AEC—and a CCD sensor. Images were obtained with 3 doses at 60 kV. The effect of metallic material also was evaluated. The contrast-to-noise ratio (CNR) for thinner steps and the low contrast value (LCV) for thicker steps were obtained. The CCD system was the best under all conditions (*P* < 0.001), although the Gray value was sensitive to the dose, and the Gray value-dose relation varied greatly. The PSP system with AEC was superior to that without AEC for the LCV (*P* < 0.001) but was inferior to it regarding the CNR (*P* < 0.001). CNR and LCV in the PSP system without AEC were not affected by the metallic plate. Intraoral digital imaging systems should be chosen according to their diagnostic purpose. PSP system with AEC may be the best for detecting molar proximal caries, whereas the PSP system without AEC may be better for evaluating small bone regeneration in periodontal disease. The CCD system provided the best performance.

## 1. Introduction

Intraoral radiography is essential for dental treatment. Silver halide film had long been used, but the introduction of intraoral digital radiography drastically changed the practices of many dental practitioners [1]. The digital system has many advantages. It reduces patient exposure, developers and fixers are no longer necessary, image quality can be adjusted for contrast and brightness, there is no degradation of films over time, and less space is required [2, 3]. A recent prevalence rate of intraoral digital radiography use was 19–30% in the United States [4]. Intraoral digital radiography is now routinely used in many university hospitals worldwide. All 29 university hospitals in Japan have adopted a digital radiography system.

Current intraoral digital radiography systems are mainly classified into two types [3]. One uses a photostimulable phosphor plate (PSP) and the other uses a charge-coupled device (CCD) sensor or a complementary metal oxide semiconductor (CMOS). The PSP system uses a plate composed of photostimulated luminescent material that can store X-ray energy and release it as luminescence. The Fuji Computed Radiography (FCR) system to be used in medicine was the first to appear, during the early 1980s [5].

The PSP is more simply called an imaging plate. CCD and CMOS sensors convert radiographic photons into electrical signals and finally into digital signals with an analog-digital converter. The images obtained from these systems do not have the same image quality (e.g., spatial resolution, pixel size, and noise), which is important diagnostically [6–11]. For example, some papers showed that, for diagnosing artificially induced external root resorption and proximal caries, images obtained by the CCD sensor were superior to those obtained using the PSP system [10, 12]. The main causes of the differences are differences in spatial resolution, the signal-to-noise ratio (SNR), or the noise of the intraoral digital systems [10, 11]. In addition, special resolution is affected in regard to detection of root fractures [8]. The objects, such as clinical or phantom images, also affect the results. The early digital systems were used mostly for research. The intraoral digital systems have made considerable progress because of rapid advances in computers, with greatly improved hardware and software. Generally distributed digital cameras are examples. The performance of the latest models is much superior to those of some years ago. The spatial resolution is >10 M pixels. Digital cameras are now used in dental and facial research with various spatial resolutions [13]. Thus, research on previous intraoral digital systems may not be applicable to the currently available systems.

The aim of the study was to evaluate the differences in the quality of images obtained from PSP and CCD sensor systems from the viewpoint of clinical application using a more precise phantom and current intraoral digital systems. We also evaluated PSP systems with and without an automatic exposure compensation (AEC) function, which corrects the contrast by adjusting the exposure range. Finally, we assessed the ability of radiopaque materials to simulate a clinical situation.

## 2. Materials and Methods

### 2.1. Subjects

**Figure 1:**
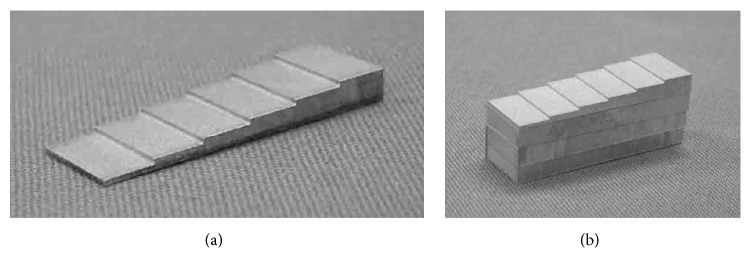
*Subjects*. (a) Aluminum step wedge with 0.5, 1.0, 1.5, 2.0, 2.5, and 3 mm thickness. (b) Aluminum step wedge and three aluminum plates with 3 mm thickness.

Aluminum step wedges of six thicknesses (0.5, 1.0, 1.5, 2.0, 2.5, and 3.0 mm) and an aluminum plate of 3 mm height were manufactured from an aluminum block of 99.9% purity (Figure 1). The two phantoms were both 10 × 30 mm. The accuracy of the height was within 0.01 mm. A lead plate measuring 5 × 8 mm with 2 mm thickness was used to simulate radiopaque materials, such as a metallic crown or an inlay in the oral cavity.

### 2.2. Radiographic System

Digital intraoral systems equipped in the department of Oral and Maxillofacial Radiology, Tokushima University Hospital, were used. The equipment included an intraoral, constant-potential, X-ray generator with total filtration of 2.0 mm aluminum (Max-DC70; Morita, Kyoto, Japan), two PSP systems [Digora Optime (Soredex, Tuusula, Finland) and VistaScan Perio (Dürr Dental AG, Bietigheim-Bissingen, Germany)], and a CCD sensor system (Megadixel; Morita).

### 2.3. Radiographic Conditions

Three aluminum step wedges were simultaneously exposed to three detector systems: the two PSP systems and a CCD sensor. A tube voltage of 60 kV and a focus-to-detector distance (FDD) of 100 cm were fixed. The exposure time was set on the basis of the clinical dose for the mandibular anterior teeth region. The exposure times were 1.6 s for the PSP systems and 0.8 s for the CCD sensor, according to the manufacturers' instructions. Exposure times were set at 0.08, 0.16, 0.40, 0.80, or 1.60 s. Doses were the standard, one-half of standard, and one-tenth of the standard clinical dose.

Three aluminum plates were added to each of the six aluminum step wedges (Figure 1(b)). Their final heights were 9.5, 10.0, 10.5, 11.0, 11.5, and 12.0 mm. The phantom was exposed under the same conditions as already described for the tube voltage and FDD. The exposure time was set as the clinical dose for the maxillary molar region. Exposure times were 0.32, 0.80, 1.60, and 3.20 s for the PSP systems, and 0.16 s was added for the CCD sensor. An experiment in which a lead plate was added was performed under the same conditions.

The measurements were repeated five times under each condition. During a preliminary experiment, a proportional relation of exposure time and dose was observed from 0.08 to 3.20 s. One second corresponds to 0.28 mGy (standard deviation 0.002).

### 2.4. Image Transfer

In clinical situations after exposure, image data are converted into Digital Imaging and Communication in Medicine (DICOM) format for each digital modality system and sent to a hospital server through Centricity PACS (GE Healthcare Japan, Tokyo) [14]. In Centricity Enterprise Web (GE healthcare Japan) server, the image data are converted into 8-bit data and delivered to a display terminal in each department. Centricity Universal Viewer (GE Healthcare Japan) was also equipped in the radiology department and all images can be observed with original bit data. Thus, all images in our hospital have 8-bit data. The original DICOM data obtained from each digital modality system in the study, however, had different image bits (Table 1). After obtaining the DICOM data, any post-image processing was not performed and all original data were directly analyzed.

### 2.5. Evaluation Method

**Figure 2:**
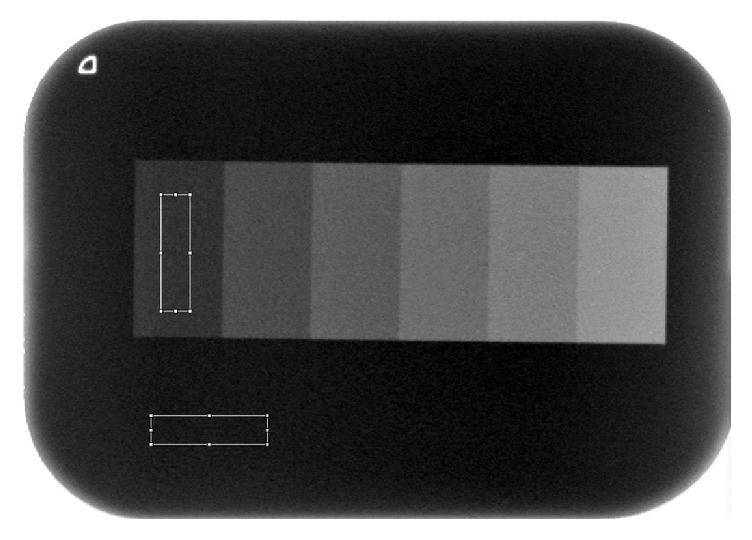
*Region of Interest (ROI)*. The ROI is 1.6 × 6.4 mm. ROIs are set at areas for each step and background.

Rectangular regions of interest (ROIs) were set on aluminum image and background (Figure 2). A size of 1.6 × 6.4 mm was determined to be the integral multiple of the pixels. Average Gray values in both ROIs were measured using ImageJ software (version 1.6.0; National Institutes of Health, Bethesda, MD, USA). Contrast for the thinner phantom (only the aluminum step wedge) was evaluated according to the contrast-to-noise ratio (CNR), which is defined by the following equation:

(1) CNR=Gray  value 0.5 mm−Gray  value BackgroundStandard  deviation Background,

where the Gray value (*x* mm) is the average for the aluminum step image at *x* mm.

The low contrast resolution for the thicker phantom (aluminum step wedges added to three aluminum plates) was evaluated according to the low contrast value (LCV). LCV was defined as the following equation:

(2) $$\begin{array}{c} LCV=\text{Gray value }\left(\;11\;mm\;\right)-\text{Gray value }\left(\;10\;mm\;\right). \end{array}$$

The 16-bit data from the VistaScan system were converted into 8-bit data, and the data bits were matched to those from the Digora Optime and Megadixel systems (Table 1). The calculation was performed using 8-bit data. The CNR and LCV were evaluated based on differences in exposure, detector type, and the presence (or not) of the lead plate. Three-way analysis of variance and multiple comparisons by Scheffe's test at a significance level of 0.05 were performed by statistical add-in software for Microsoft Excel (version1.13; Social Survey Research Information Co., Ltd., Tokyo, Japan).

## 3. Results

### 3.1. Gray Values for the Thinner Phantom

**Figure 3:**
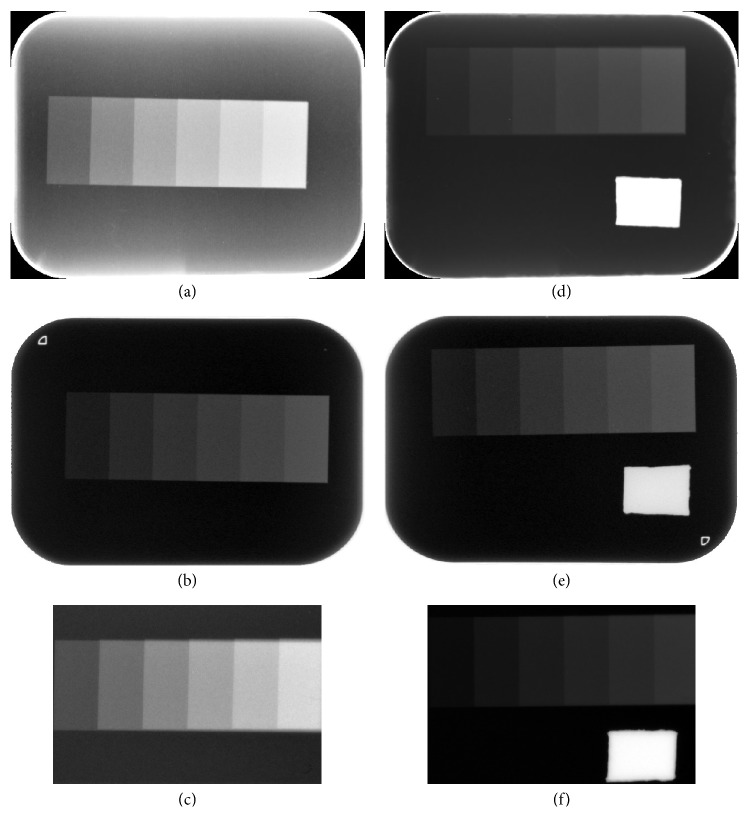
*Images of Thinner Aluminum Step Wedges Obtained by a Photostimulable Phosphor Plate (PSP) System and a Charge-Coupled Device CCD Sensor*. A lead plate is placed at d, e, and f images. (a, d) Digora images. (b, e) VistaScan images. (c, f) Megadixel images.

With the thinner phantom, images by the Digora and Megadixel systems had visually similar image contrast, whereas the Gray level and contrast of the images by VistaScan decreased using the standard dose. Adding a lead plate caused the Gray level to increase and the contrast to decrease in the Digora and Megadixel images, whereas the Gray level for the VistaScan images hardly changed (Figure 3, Table 2).

**Figure 4:**
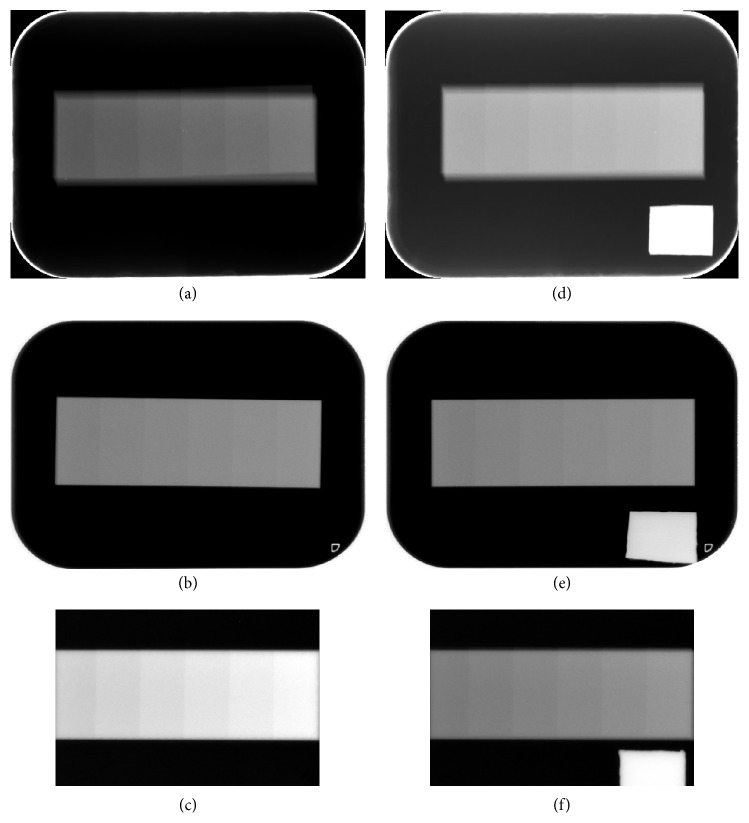
*Images of Thicker Aluminum Step Wedges by a PSP System and a CCD Sensor*. A lead plate is also placed in the d, e, and f images. (a, d) Digora images. (b, e) VistaScan images. (c, f) Megadixel images.

Overall, Gray values were higher with increased aluminum thickness. There was an almost linear relation between the Gray values and the thickness. With the Digora system, however, the gradient between 0.5 mm thickness and background was slight, and the linear relation collapsed. There were no changes in Gray values with the various exposure doses. With the VistaScan system, similar graphic shapes were shown at all doses and regardless of the presence of the lead plate. In contrast, with the Megadixel system, the gradient changed dramatically at around the point of 1.5 mm thickness without the lead plate. When the lead plate was added, the gradient decreased. In addition, the Gray value decreased with increasing doses (Figure 4, Table 2).

### 3.2. Gray Values for the Thicker Phantom

**Figure 5:**
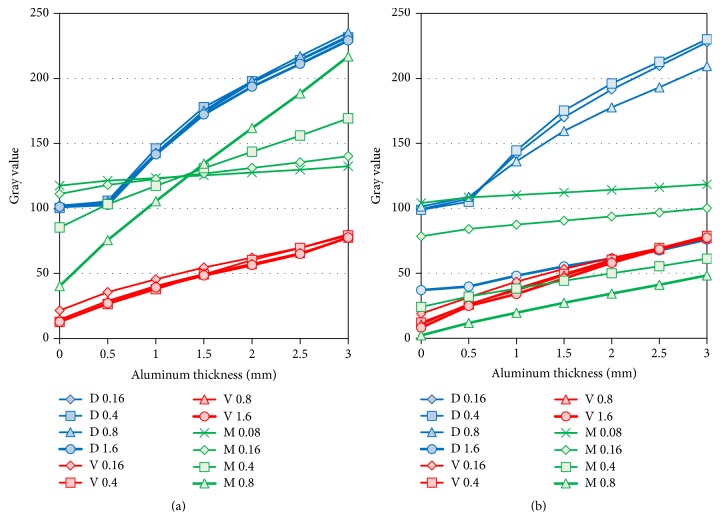
*Comparison of Gray Values for Thinner Aluminum Step Wedges*. D, V, and M indicate the Digora, VistaScan, and Megadixel detectors' values. Bold lines of the detectors (D1.6, V1.6, and M0.8) show the Gray levels at standard exposure. (a) Exposure condition with the aluminum step wedge alone. (b) Exposure condition with the aluminum step wedge and the lead plate.

With the thicker phantom, the image contrast decreased compared to that for the thinner phantom for all systems at the standard dose. When the lead plate was added, the Gray value for the Megadixel system images decreased, whereas that for the Digora system images increased. Adding the lead plate hardly changed the Gray value for the VistaScan images (Figure 5, Table 3).

**Figure 6:**
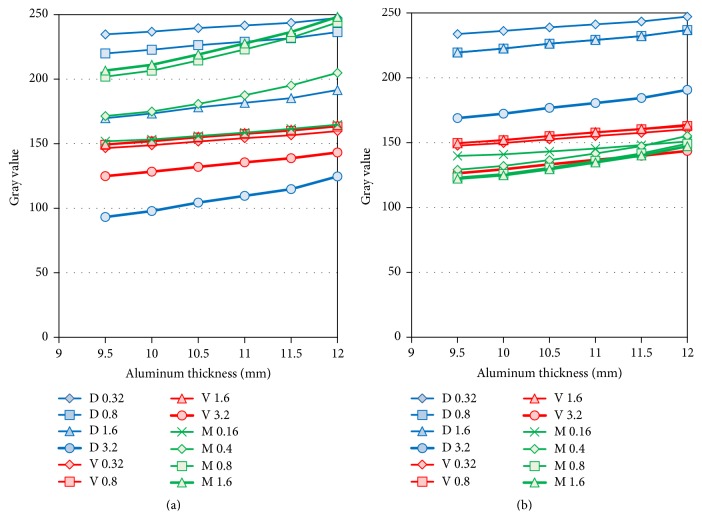
*Comparison of Gray Values for Thicker Aluminum Step Wedges*. D, V, and M indicate the Digora, VistaScan, and Megadixel detectors' values. Bold lines of the detectors (D3.2, V3.2, and M1.6) show the Gray levels at standard exposure. (a) Exposure condition with only the aluminum step wedge. (b) Exposure condition with the aluminum step wedge and the lead plate.

The Gray value for the thicker phantom increased with increasing aluminum thickness overall, but the gradient was slight compared with that for the thinner phantom. With the Digora system, the Gray value decreased with increasing doses, whereas with the Megadixel system it increased. Gray values with the VistaScan system changed slightly. When the lead plate was added, the Gray value increased at the standard dose with the Digora system, decreased at all doses with the Megadixel system, and hardly changed at any of the doses with the VistaScan system (Figure 6, Table 4).

### 3.3. CNR

**Figure 7:**
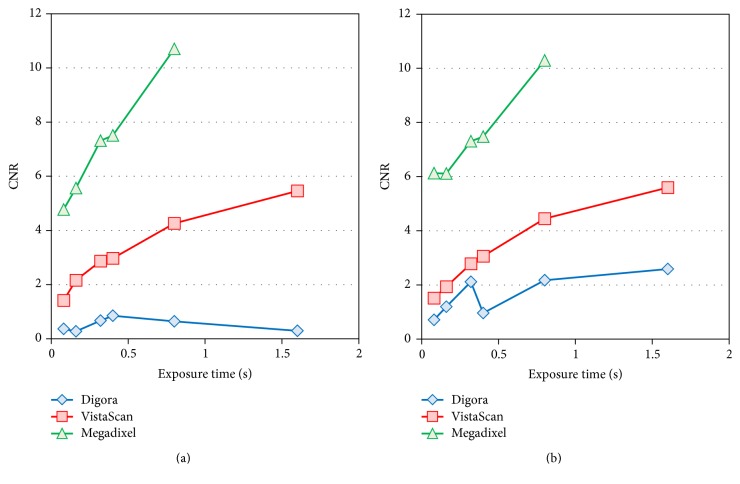
*Comparison of the Contrast-to-Noise Ratio (CNR) for the Thinner Aluminum Step Wedge*. (a) Exposure condition with only the aluminum step wedge. (b) Exposure condition with the aluminum step wedge and the lead plate.

The CNR was highest with the Megadixel system and lowest with the Digora system at all doses with or without the presence of the lead plate (*P* < 0.001). CNRs for the Megadixel and VistaScan systems increased with increasing doses (*P* < 0.001). In contrast, with the Digora system, the CNR changed very little without the lead plate but increased with it (*P* < 0.05) (Figure 7, Tables 2 and 4).

### 3.4. Low Contrast Value

**Figure 8:**
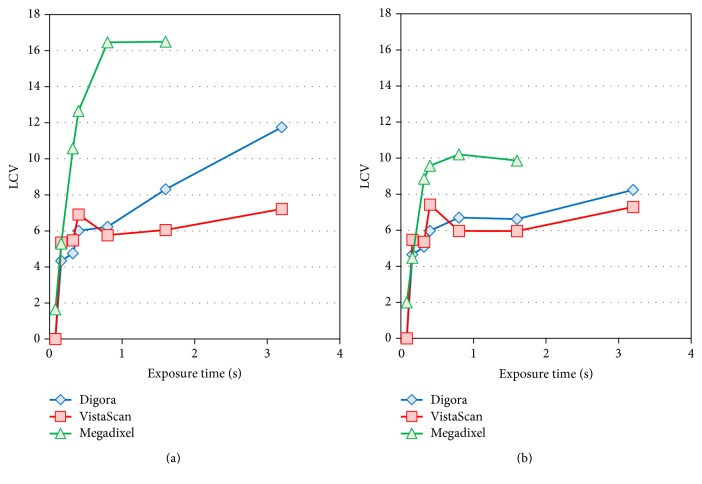
*Comparison of the Low Contrast Value (LCV) for the Thicker Aluminum Step Wedge*. (a) Exposure condition with only the aluminum step wedge. (b) Exposure condition with the aluminum step wedge and the lead plate.

The low contrast value (LCV) was highest with the Megadixel system and lowest with the VistaScan system without the lead plate (*P* < 0.001). Adding the lead plate caused the LCVs to decrease with the Megadixel and Digora systems (*P* < 0.001), whereas with the VistaScan system the LCV did not change. With the Megadixel system, the LCV was almost constant at doses of more than one-half of the standard dose with or without the lead plate. With the VistaScan system, the LCV decreased at doses that were less than the standard dose (*P* < 0.05). With the Digora system, the LCV decreased with decreasing doses, with or without the presence of the lead plate (*P* < 0.001) (Figure 8, Tables 3 and 5). The presence of the lead plate decreased the LCV, which was the opposite to the reaction of the CNR.

## 4. Discussion

Experimental errors in this field are thought to be caused by age-related degradation of the system, instability of the exposure dose, and the precision of the phantom. Although age-related degradation of the digital system affects sensitivity and contrast, it has been reported that the decrease is slight, with only a <1% decrease in signal intensity after 2 years of use [15]. According to the manufacturer's instructions, maintenance and inspection of FCR systems using PSP are recommended because the sensitivity decreases due to the age-related degradation of the laser tube used for reading. All of the intraoral digital systems used in the study had been equipped around the same time and had undergone periodic maintenance and inspection. Also, when a PSP was scratched, it was replaced by a new PSP. Thus, we believe that the results of this study have not been influenced by the degradation of systems including the PSP.

The phantom used in the study was an aluminum step wedge and a plate. Although there are numerous reports of experiments conducted to assess detectors using an aluminum step wedge, its accuracy has been little considered, and a step wedge in steps of 1 mm thickness is generally used [16]. Because a thickness increase in 0.5 mm increments with aluminum steps was used in the study, it revealed new findings. This very small thickness increase is necessary for a detailed evaluation of detectors.

Concerning the radiopacity of materials, the International Organization for Standardization has stated that radiopacity is evaluated using the aluminum step wedge technique with a range of 0.5–5.0 mm thickness at 0.5 mm increments at an accuracy of >98% purity and 0.05 mm variation in thickness [17]. Any error would greatly affect the evaluation of very slight differences in signal intensity. In the present study, the precision of 0.01 mm at 0.5 mm aluminum thickness was achieved by a special sandblasting process. Thus, the possibility of an error in thickness is <1%. Another cause of error is dose instability. The exposure dose was highly stable and reproducible, with an error of <1% because we used a high-frequency generator so it maintained constant high voltage. In addition, exposure was performed simultaneously with the three detectors, with no differences in the doses among the detectors. Aluminum step wedges with the highest precision made the simultaneous exposure possible. Based on these findings, the total error was <5% when considering systematic degradation, mechanistic accuracy of the phantom, and exposure dose variation. The overall experimental accuracy was considered to be quite high.

Images obtained from a detector could be classified into four groups by two phantoms (thinner and thicker) with or without a lead plate. These images had very different contrast among the three detectors, although the imaging conditions were the same as those used in our university hospital. This point showed that it would be necessary to adjust the contrast and brightness of images used for diagnosis in the clinical setting. The AEC automatic exposure compensation (AEC) function in the Digora and Megadixel systems could help shorten the time to diagnosis. Using the AEC function improved image contrast with a lead plate, as shown in Figures 3 and 4. The image contrast provided by VistaScan without AEC, however, did not depend on the presence of the lead plate. This finding indicated that radiopaque materials (e.g., metallic crowns, inlays) greatly affect image contrast, depending on the system used, so the diagnostic results may vary from one system to another.

In Figure 5 the relationship between Gray value and aluminum thickness showed linearity in the VistaScan and the Megadixel, but that of the Digora showed convex. The difference of the curves between the Megadixel and the Digora was considered to be due to function of the logarithm of exposure by software. And higher exposure at 0 or 0.5 mm aluminum thickness was considered not to be discriminated in the Digora because the Gray value at higher exposure was compressed by the logarithmic conversion and the slight difference disappeared. The function of the logarithm has been installed from old model of Digora system [18]. The AEC function, however, is inadequate when compared to the exposure data recognizer (EDR) in FCRs used in the medical field. EDR can automatically adjust the latitude and sensitivity when reading an imaging plate and display an optimal image for histographic analyses [5]. Moreover, not software but hardware, a logarithmic amplifier, is equipped in FCR system and the linear relationship between signal level and exposure is maintained. The authors expect that dental manufacturers will continue to develop software for intraoral digital systems and introduce a function such as EDR into dentistry.

The digital systems also produce different bit image data. The 16-bit image data derived from the VistaScan system were converted into 8-bit data by linear transformation. The bit difference reduces discrimination of slight contrast. The bit depth of reading is 14 bits, but 8-bit data are output by the Digora and Megadixel systems because the AEC function, which adjusts density automatically, is incorporated into these systems, and the exposure range is adjusted from a 14-bit Gray value to an 8-bit Gray value. In the Digora system, the method is not a simple linear transformation. As shown in Figure 5, the line is slightly curved and is broken at the point of 0.5 mm aluminum thickness. In the Megadixel system, the linearity is maintained. The optimal Gray level range, including the target, is automatically selected and is converted into 8-bit data. That is, automatic adjustments of the density and contrast are included to delete noise that is irrelevant to an object. Concerning image bit depth, previous studies reported that higher image bit depth was superior to lower image bit depth [16, 19]. These early digital imaging instruments did not have the AEC function. When the AEC function is provided in all systems, the results could be different. It was reported that radiologists generally preferred 8-bit displays, although the higher Gray scale resolution resulted in more complete visualization of image information. The radiologists, however, partially judged it as a lack of sharpness and contrast [20]. Thus, it was difficult to utilize higher-bit displays (e.g., 14- or 16-bit data). Some recent studies evaluated the importance of a DICOM-calibrated, 8-bit Gray scale monitor [21]. All image data were transferred to 8-bit data by linear transformation because the Picture Archiving Communication System in Japanese hospitals generally uses 8-bit data images. The influence of the conversion method, however, also should be evaluated in the future.

Numerous studies have compared PSP and CCD sensor systems. A CCD sensor is superior to PSP in physical performance, but no significant difference is seen in the clinical setting. One study reported that, based on the results of visual assessment of caries in extracted teeth, there was no difference between the PSP system, CCD system, and radiographic film [7]. The experimental situation is different from a clinical situation in that there are no scattering substances such as a cheek or tongue and no radiopaque dental fillings. As a result, slight contrast can be difficult to detect. One of the PSP systems used in this study, the Digora system, had the AEC function and showed a clear difference with or without the presence of the lead plate. The influence of the AEC function and radiopaque materials on caries detection should be evaluated in future research using this method. In contrast, one study reported that the CCD system was superior to the PSP system regarding visual assessment of extracted teeth with fractures [8]. The authors concluded that it was due to spatial resolution. In the present study, the contrast provided by the CCD system was best under all conditions. The difference of slight contrast, rather than spatial resolution, was considered to be the cause. A study that changes the spatial resolution in a CCD system could clarify the cause.

A recent report supported our results [9]. Using extracted fractured teeth, the difference in detection between the PSP system, CCD system, and radiographic film was evaluated. The authors showed that there was a significant difference between them and that the CCD system was the best. Moreover, an experiment closer to the clinical condition was reported [10]. The aim of the study was to find any differences in detecting tooth fractures using PSP, CCD, and radiographic film. The results indicated that the CCD system and film were significantly superior to the PSP system. It was concluded that the differences depended on spatial resolution and the SNR. However, early systems were used, and their levels of performance were inferior to those of the current systems. In fact, most previous research had been performed using the early systems, without ideal conditions such as radiopaque materials and a scattered substance. The results of those studies may not correspond to the experimental results using a currently available system. The results of our study are considered to be valuable because the experiment was performed using current PSP and CCD systems and a highly precise phantom.

Future studies should include research on image contrast using lead foil. The effect of lead foil on intraoral digital detectors has been reported [8]. Adding lead foil to the back of the detector can reduce patient exposure by 32% when using a PSP system and by 59% with a CMOS sensor. By putting a copper filter on the aluminum filter of the X-ray tube, the entrance dose decreases by 50%, and the effective dose decreases by 40%. Lead foil has been generally used on the back of intraoral film to reduce patient exposure and to prevent backscatter. Based on the results, evaluating the effect on image quality, such as contrast, should be another goal.

## 5. Conclusions

This study more accurately reflected the clinical setting during intraoral digital radiography than a previous experiment using aluminum wedge steps. It was shown that there were some differences between the CCD and PSP systems and between different PSP systems. There are two valuable measures that can be used clinically. One is the CNR, which can discriminate the low contrast of thinner objects, such as minute bone regeneration during periodontal treatment. The other is the LCV, which can discriminate low contrast of thicker objects, such as molar proximal caries. The CCD system performed best, but the operator must pay special attention to avoid overdoses. In PSP systems, the results depend on the use of software, such as the AEC function and data bit depth. Intraoral digital systems could serve an important diagnostic purpose in the clinical setting. Because this research was designed as an in vitro study, clinical studies are required in the future.

## Figures and Tables

**Table 1:** Specifications of intraoral digital detectors.

	Digora	VistaScan	Megadixel
Sensor type	PSP	PSP	CCD
Size (mm)	41 × 31	39 × 28	30 × 20
Matrix size	640 × 484	787 × 553	1500 × 1000
Pixel size (mm)	0.064	0.05	0.02
Image format	DICOM	DICOM	DICOM
Input Gray level (bits)	14	16	14
Output Gray level (bits)	8	16	8

**Table 2:** Contrast-to-noise ratio for each detector.

Dose	Digora		VistaScan		Megadixel	
	Pb (−)	Pb (+)	Pb (−)	Pb (+)	Pb (−)	Pb (+)
	mean (SD)	mean (SD)	mean (SD)	mean (SD)	mean (SD)	mean (SD)
Standard	0.31 (0.48)	2.63 (1.07)	5.46 (0.60)	5.63 (1.42)	10.70 (0.06)	10.28 (0.09)
Half	0.64 (0.47)	2.23 (1.27)	4.26 (0.26)	4.48 (0.98)	7.51 (0.10)	7.48 (0.19)
One-tenth	0.27 (0.29)	1.20 (0.52)	2.16 (0.17)	1.94 (0.07)	4.79 (0.20)	6.11 (0.33)

Each dose for CCD (Megadixel) is half of that for PSP (Digora and VistaScan).

Pb shows the existence of lead during exposure. (−) and (+) mean without and with lead.

**Table 3:** Low contrast value for each detector.

Dose	Digora		VistaScan		Megadixel	
	Pb (−)	Pb (+)	Pb (−)	Pb (+)	Pb (−)	Pb (+)
	mean (SD)	mean (SD)	mean (SD)	mean (SD)	mean (SD)	mean (SD)
Standard	11.75 (0.91)	8.23 (0.94)	7.22 (0.10)	7.29 (0.19)	16.50 (0.15)	9.86 (0.19)
Half	8.31 (0.63)	6.62 (0.74)	6.05 (0.22)	5.95 (0.15)	16.50 (0.07)	10.20 (0.16)
One-tenth	4.76 (0.66)	5.08 (0.67)	5.48 (0.18)	5.36 (0.27)	5.30 (0.23)	4.47 (0.09)

Each dose for CCD (Megadixel) is half of that for PSP (Digora and VistaScan).

Pb shows the existence of lead during exposure. (−) and (+) mean without and with lead.

**Table 4:** Three-way ANOVA and multiple comparison of the CNR.

Source of variation	*P* value (ANOVA)	Multiple comparison	*P* value
Detector	<0.001	Megadixel > VistaScan > Digora	<0.001
Lead	<0.001	With > without	<0.001
Exposure time	<0.001	Standard > half > one-tenth	<0.001
Detector*∗*lead	<0.001	Digora	<0.001
Detector*∗*exposure time	<0.001	Digora, VistaScan, Megadixel	<0.001
Lead*∗*exposure time	0.960		

ANOVA: analysis of variance; CNR: contrast-to-noise ratio.

**Table 5:** Three-way ANOVA and multiple comparison of the low contrast value.

Source of Variation	*P* value (ANOVA)	Multiple comparison	*P* value
Detector	<0.001	Megadixel > Digora > VistaScan	<0.001
Lead	<0.001	Without > with	<0.001
Exposure time	<0.001	Standard > half > one-tenth	<0.001
Detector*∗*lead	<0.001	Digora, Megadixel	<0.001
Detector*∗*exposure time	<0.001	Digora, VistaScan, Megadixel	<0.001
Lead*∗*exposure time	<0.001	Standard, half	<0.001
